# UHTC ceramics derived from B_4_C and MAX phases by reactive sintering

**DOI:** 10.1038/s41598-025-18661-z

**Published:** 2025-09-29

**Authors:** Dawid Kozień, Adrian Graboś, Katarzyna Pasiut, Magdalena Ziąbka, Leszek Chlubny, Marcin Wójtowicz, Wojciech Banaś, Marek Grabowy, Zbigniew Pędzich

**Affiliations:** 1 Faculty of Materials Science and Ceramics, Department of Ceramics and Refractory Materials, AGH University of Krakow, 30 Mickiewicz Av, 30-059 Krakow, Poland; 2 Advanced Institute for Materials Research, Tohoku University, Tohoku Forum for Creativity, 2–1–1 Katahira, Aoba-ku, Sendai, 980–8577 Japan; 3 Institute of Power Engineering, Ceramic Branch CEREL, 1 Techniczna St, 36-040 Boguchwała, Poland

**Keywords:** Boron carbide, Ceramic matrix composites, MAX phases, Ultra-high temperature ceramics, Materials science, Ceramics

## Abstract

In this work, the influence of three MAX phases (Ti_3_SiC_2,_ Ti_2_AlC, and Cr_2_AlC) on the densification and final properties of dense composites from Ultra-High Temperature Ceramics (UHTC) family was studied. Addition of the MAX phases resulted in the formation of secondary boride phases due to chemical reactions between boron carbide and MAX phase. Mentioned phases were utilized for reduction of sintering temperature of the final composite material. It decreased the sintering temperature up to 800 °C when compared to pure B_4_C. Additionally, the phase composition and derivative mechanical properties were investigated to evaluate differences between final composite materials. All obtained materials remarkably increased their fracture resistance (K_IC_) from 33 to 100%. The mechanical properties of B_4_C were either retained (Ti_3_SiC_2_) or decreased in terms of Vickers hardness and Young’s Modulus (Ti_2_AlC and Cr_2_AlC). Systems with Ti_3_SiC_2_ appeared to possess significant potential for application, also when compared to UHTC systems of similar purpose.

## Introduction

Ultra-high-temperature ceramics (UHTCs) are a group of ceramic materials that is suitable for operation at very high temperatures, owing to their relatively high melting points, reaching values above 3000 °C^1^. Most often, UHTCs are characterized mainly by their high thermal stability, high thermal conductivity, high hardness, and low density^2–4^. Therefore, these materials have recently been considered as potential for used in extreme environments as resources for aircraft, spacecraft, nuclear reactors and others prospective applications^5^. Among these, boron-based systems have been studied because of their mechanical resistance, corrosion resistance and melting temperatures above 2500 °C. There are two types of boron-based UHTC carbides (e.g.B_4_C) and borides (for example, TiB_2_)^6–8^.

The use of the described materials is limited by two factors: the low relative density of the final product and the high sintering temperature. Relative density is very important when any properties of covalent materials are considered. Both thermal and mechanical resistance are highly influenced by the amount of porosity introduced by the specific manufacture technology, which is an important research aspect in many reports^9–12^. High sintering temperatures are important in terms of industrial application of UHTCs materials due to possible reduction of energy consumption, which is very desired in the incoming years^13^. Several additives were considered in order to reduce the sintering temperatures and simultaneously improve density of borides and boron-based carbides. Those included: Al_2_O_3_, MoSi_2_, C, Y_2_O_3_, bringing various results and materials with mixed phases that influence their final properties^14–17^.

Simultaneously, a relatively new commercialized group of ceramic materials, called MAX phases, possess similar properties, but their working temperatures are about 1000 °C lower than those of UHTCs. Additionally, their unique crystallographic structure is resulting in their relatively easy mechanical treatment and metal-like properties^18,19^. They are composed of transition metals (M), A-group elements (A), and carbon or nitrogen (X), and have been extensively studied as potential construction or coating materials^20,21^. Their composition is important for boron-based ceramics because typically utilized MAX phases are composed of boron compound-forming elements (for example, Ti, C) or their valid sintering additives (for example, Mo, Cr). Therefore, such materials could be expected to form promising ceramic matrix composite (CMC) microstructures, characterized by low density, low sintering temperature and preserved mechanical properties, making them desirable for the industrial application of UHTCs, and competitive option in comparison to other ceramic-based composites^22–24^.

The aim of this study was to synthesize a multiphase UHTCs material based on B_4_C and popular MAX phase precursors, namely Ti_3_SiC_2_, Ti_2_AlC, and Cr_2_AlC. The influence of MAX phase additives on the sintering process, density, microstructure and mechanical properties of the final product was studied using several evaluation methods to determine their potential industrial applicability.

## Materials and methods

The sintering was performed utilizing following reactants: 99.0% purity B_4_C powders provided by H.C STARCK (Goslar, Germany) and powders of Ti_3_SiC_2_, Ti_2_AlC and Cr_2_AlC, which were obtained by milling of previously self-propagation high-temperature synthesized (SHS) MAX phases according to procedure described in^25^. Samples were wet homogenized using isopropanol for 30 min in a mortar, with the compositions presented in Table 1. A numbers in the name of samples indicate the amount of MAX phase added to the mixture in weight percents.

**Table 1:** Composition of samples.

Sample	B_4_C [%]	Ti_3_SiC_2_ [%]	Ti_2_AlC [%]	Cr_2_AlC [%]
B_4_C_Ti_3_SiC_2__5	95	5	-	-
B_4_C_Ti_3_SiC_2__10	90	10	-	-
B_4_C_Ti_3_SiC_2__15	85	15	-	-
B_4_C_Ti_3_SiC_2__20	80	20	-	-
B_4_C_Ti_2_AlC_5	95	-	5	-
B_4_C_Ti_2_AlC_10	90	-	10	-
B_4_C_Ti_2_AlC_15	85	-	15	-
B_4_C_Ti_2_AlC_20	80	-	20	-
B_4_C_Cr_2_AlC_5	95	-	-	5
B_4_C_Cr_2_AlC_10	90	-	-	10
B_4_C_Cr_2_AlC_15	85	-	-	15
B_4_C_Cr_2_AlC_20	80	-	-	20

Subsequently, the obtained samples underwent uniaxial pressing, which was performed in two stages to reduce oxygen in the form and in-between grains. In the first stage, the prepared powders were pressed uniaxially at a pressure of 30 MPa and subjected to isostatic pressing at a pressure of 100–200 MPa. The obtained round tablets were pressureless sintered with a heating rate of 10 °C/min in a dilatometer to determine the sintering temperature basing on sample shrinkage. After sintering density of samples was calculated basing on their mass and geometrical dimensions.

Young’s Modulus was determined using an ultrasonic flaw detector (Olympus Epoch 650, Hamburg, Germany), measuring the speed of the wave traversing along the sample height (short edge) based on the following formula:

1 $$\:E = v_{t}^{2} \cdot\rho \:$$

where: *E* – Young’s Modulus; ρ – sample density; *v*_*t*_ – transverse wave velocity;

Hardness (*HV*) was determined using a Vickers hardness testing machine (Future-Tech FM-700 hardness tester, Kawasaki-City, Japan), performing three tests for each sample with a 9.81 N load for 10 s.

2 $$\:HV = 0.1891\:\cdot\frac{F}{{d^{2} }}$$

where: *d* – mean diagonal of indentation (µm); *F* –applied load (N).

The method of measuring the Palmqvist crack length in the Vickers hardness test was used to calculate the fracture resistance K_Ic_ based on the Nihara–Moreno–Hasselmann formula^26,27^.

3 $$\:K_{{1C}} = 0.0309\:\cdot(\frac{E}{{HV}})^{2} \cdot\frac{F}{{C^{{3/2}} }}\:$$

Where: K_Ic_—the fracture toughness, HV- hardness, E—Young modulus, *F* –applied load (N), C = l + d/2; l is an arithmetic mean a value of the measured cracks; and d is an arithmetic mean value of the measured diagonals of indentation.

Four tests were performed, and 9.81 N force was used, respectively. Phase analysis was performed using a monochromatic beam of emitted wavelength equal to Cu_Kα_ (1,5405 Å) and conducted with a PANalytical Empyrean X-ray diffractometer (Almelo, The Netherlands). X-pert HighScore software was used for result analysis. Powder homogenization and sample microstructure were investigated using Scanning Electron Microscopy (Nova NanoSEM 200 by FEI) and Energy Dispersive Spectroscopy (EDS by EDAX, Eindhoven, The Netherlands) for elemental mapping. Samples observed using SEM/EDS where polished using discs up to the 1200 gradation and subsequently using polishing cloth (1 μm) with diamonds 1 μm dispersion. Elemental mapping was done using backscattered electrons (BSE) mode.

## Calculations

Ab-initio calculations regarding the formation of secondary phases were conducted preceding the sintering process, based on previously obtained results and sources^28^. Their results were presented below:

B_4_C_Ti_3_SiC_2_ group samples.

4 $$8B_{4} C + {\text{ }}15Ti_{3} SiC_{2} = {\text{ }}16TiB_{2} + {\text{ }}3Si_{5} C_{3} + {\text{ }}29TiC$$

B_4_C_ Cr_2_AlC group samples.

5 $$13B_{4} C + {\text{ }}30Cr_{2} AlC = {\text{ }}26AlB_{2} + {\text{ }}Al_{4} C_{3} + {\text{ }}20Cr_{3} C_{2}$$

B_4_C_Ti_2_AlC group samples.

6 $$2B_{4} C + {\text{ }}3Ti_{2} AlC = TiB_{2} + {\text{ }}3AlB_{2} + {\text{ }}5TiC$$

It was expected for every sample to contain a certain mixture of carbide and boride phases, creating a similar microstructure to previously referred composites using B_4_C as a matrix. XB_2_ phase was expected to be present in every structure, ranging from the dominant reinforcement phase (B_4_C_Cr_2_AlC group samples) to the least abundant phase (B_4_C_Ti_2_AlC group samples). Other reinforcement phases would be mostly derivative carbides, with exception of AlB_2_ created in B_4_C_Ti_2_AlC.

## Results

Generally, in all investigated cases sintering processes had character of reactive sintering. In each case boron carbide reacted with MAX phase and the final material was a typical particulate composite. Depending on a type and amount of applied MAX phase, the level of densification, a phase composition and microstructure of resulting composites were distinctly different.

### Sintering process

**Fig. 1:**
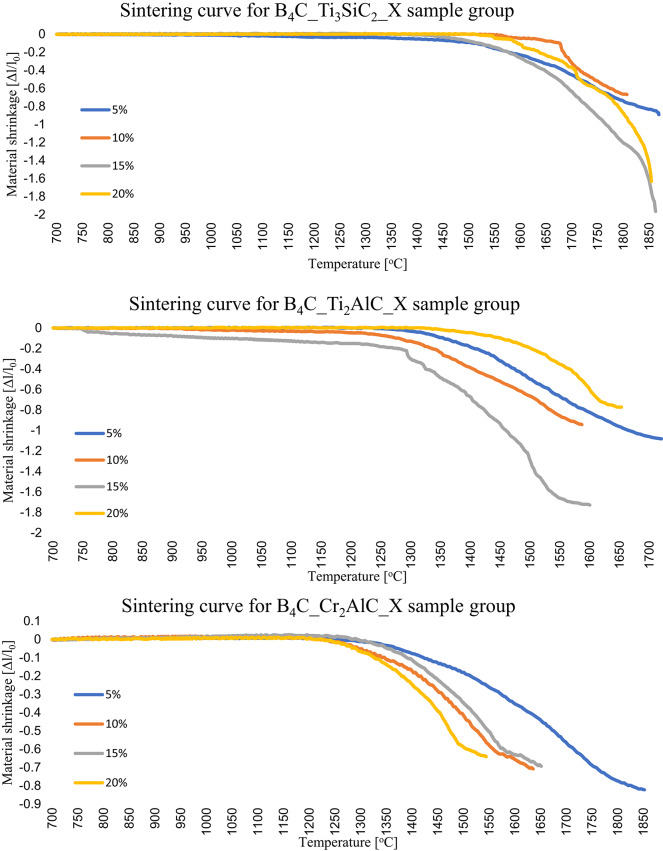
Sintering curves of samples obtained in each material group.

Conducting the sintering process in dilatometer allowed us to determine shrinkage kinetics of the samples and determine the material’s sintering onset temperature. In Fig. 1, the sintering curves of each sample group were presented. The beginning of the sintering process is observed by substantial material shrinkage, noted as sample length change (Δl) in comparison to initial length (l_0_). For samples with the addition of Ti_3_SiC_2_ the lowest temperature initiating the sintering process occurred for the sample of B_4_C_Ti_3_SiC_2__15 (1450 °C) while the highest temperature equaled 1580 °C and was recorded for B_4_C_Ti_3_SiC_2__10 sample. Simultaneously, sample with 15 wt% addition of Ti_3_SiC_2_ was the sample with lowest overall shrinkage and sample with 10 wt% addition of Ti_3_SiC_2_ had highest overall shrinkage. Samples with the addition of Ti_2_AlC had the lowest sintering temperature recorded for B_4_C_Ti_2_AlC_10 and B_4_C_Ti_2_AlC_15 samples (1290 °C) and highest for B_4_C_Ti_2_AlC_20 sample, recorded at 1350 °C. Similarly to the samples with addition of Ti_3_SiC_2_, the highest overall shrinkage was recorded for the samples with lower sintering temperatures and lowest for the samples with higher sintering temperature. Lastly, samples with the addition of Cr_2_AlC started their sintering process at similar temperatures, but the lowest sintering onset temperature is characterized by the samples of B_4_C_Cr_2_AlC_10 and B_4_C_Cr_2_AlC_20, noted at about 1260 °C. Following samples: B_4_C_Cr_2_AlC_5 and B_4_C_Cr_2_AlC_15 had similar recorded temperatures of 1300 °C and 1320 °C respectively. In contrary to the previous systems, the lowest starting temperature was not an indicator of the highest overall shrinkage. Therefore, a B_4_C_Cr_2_AlC_20 sample was noted to be of lowest sintering temperature and lowest overall shrinkage simultaneously.

### Sample density and young’s modulus

Measured sample densities, wave velocities, and Young’s Modulus were collected in Table 2. It was revealed that the addition of MAX phase precursors allows for the reduction of measured density in comparison to pure B_4_C’s literature density. The measured density reduction was notable in each sample group, with the lowest value for samples with additions of Ti_3_SiC_2_ and the highest for samples with additions of Cr_2_AlC, reaching about 0,50 g/cm^3^ reduction difference in the peak. However, due to the sample partial porosity present in the secondary phase (4.3.2.), this reduction should be discussed in context of measured Young’s Modulus. It was clear, that with the increasing addition of each MAX phases, the Young’s Modulus was either decreasing (samples B_4_C_Ti_3_SiC_2__5–20, B_4_C_Ti_2_AlC_20, and B_4_C_Cr_2_AlC_10) or was preserved at the same level. Therefore, although measured density was reduced, particular reductions can be related to the increasing porosity within the samples. A notable example of this would be sudden decrease of Young’s Modulus for B_4_C_Ti_2_AlC_20 sample group. Although the measured density of this sample group is comparable to the rest samples with the additions of Ti_2_AlC MAX phase, it is a much more porous group. Another interesting example is abnormal reduction of B_4_C_Ti_3_SiC_2__15 sample group measured density in comparison to the rest sample groups with Ti_3_SiC_2_ additions, while maintaining Young Modulus compared to the B_4_C_Ti_3_SiC_2__20. This may indicate that the peak values of MAX phases additions for both groups were in the range between 10 and 15 wt%, but further process optimization has to be done, to determine this peak accurately. Additionally, when compared to commercially available B_4_C and previously obtained results regarding similarly obtained, different composites^29,30^, only B_4_C_Ti_3_SiC_2__5 and B_4_C_Ti_3_SiC_2__10 samples possessed similar Young’s Modulus, while other samples noted a decrease of it. This is yet another indicator that the proposed process is largely susceptible to the possible added porosity of the final materials which could affect final properties^30–34^.

**Table 2:** Measured densities, wave velocities in transverse pass and young’s modulus of samples.

Sample	Measured density [g/cm^3^]	Wave velocity [m/s]	Young’s Modulus [GPa]
Ti_3_SiC_2_			
B_4_C_Ti_3_SiC_2__5	2.38	13,682	444
B_4_C_Ti_3_SiC_2__10	2.45	12,720	396
B_4_C_Ti_3_SiC_2__15	2.06	12,884	341
B_4_C_Ti_3_SiC_2__20	2.45	11,730	336
Ti_2_AlC			
B_4_C_Ti_2_AlC_5	2.14	12,660	343
B_4_C_Ti_2_AlC_10	2.19	12,482	340
B_4_C_Ti_2_AlC_15	2.36	12,176	350
B_4_C_Ti_2_AlC_20	2.26	11,664	308
Cr_2_AlC			
B_4_C_Cr_2_AlC_5	1.96	12,508	307
B_4_C_Cr_2_AlC_10	2.06	11,654	279
B_4_C_Cr_2_AlC_15	2.07	11,584	278
B_4_C_Cr_2_AlC_20	2.36	10,928	282

### Phase analysis

#### XRD diffraction

In order to determine the exact phase composition of obtained UHTCs, XRD diffraction was performed. Results were presented collectively in Table 3. The Rietveld method was employed to determine the proportion of crystalline phases quantitatively. Following the aggregation of data from three samples per composite, the findings were averaged.

**Table 3:** Phase composition of each sample by groups.

Sample	B_4_C [%]	TiB_2_ [%]	TiC [%]	Si_5_C_3_ [%]	AlB_2_ [%]	CrB_2_ [%]	Cr_3_C_2_ [%]
Ti_3_SiC_2_							
B_4_C_Ti_3_SiC_2__5	92.5	7.0	0.3	0.3	0	0	0
B_4_C_Ti_3_SiC_2__10	88.6	8.1	0.6	2.7	0	0	0
B_4_C_Ti_3_SiC_2__15	86.9	12.1	1.0	0.1	0	0	0
B_4_C_Ti_3_SiC_2__20	83.0	14.7	1.4	0.9	0	0	0
Ti_2_AlC							
B_4_C_Ti_2_AlC_5	96.5	2.0	0	0	1.5	0	0
B_4_C_Ti_2_AlC_10	92.7	3.9	0	0	3.3	0	0
B_4_C_Ti_2_AlC_15	90.3	6.0	0	0	3.7	0	0
B_4_C_Ti_2_AlC_20	86.3	6.8	0	0	6.9	0	0
Cr_2_AlC							
B_4_C_Cr_2_AlC_5	97.2	0	0	0	0.1	2.7	0
B_4_C_Cr_2_AlC_10	91.6	0	0	0	0.2	8.2	0
B_4_C_Cr_2_AlC_15	80.7	0	0	0	0.4	18.9	0
B_4_C_Cr_2_AlC_20	80.0	0	0	0	1.0	19.0	0

It was determined that the addition of Ti_2_AlC and Cr_2_AlC MAX phase precursors to the B_4_C resulted in the creation of two secondary boride phases as predicted. The amount of those phases did not increase proportionally. In B_4_C_Ti_2_AlC sample group the formation of TiB_2_ was visibly preferred due to the enthalpy of such a reaction. Only when the Al addition was sufficiently high, the formation of AlB_2_ followed. Therefore, it can be noted that the amount of AlB_2_ can only be lower or equal to TiB_2_. Samples of B_4_C_Cr_2_AlC followed a similar pattern, but CrB_2_ was much more strongly preferred, and Al was almost missing in the final composition, although ab-initio calculations would suggest otherwise. In the sample group of B_4_C_Ti_3_SiC_2_, similarly to Al in previous groups, Si-based phases were mostly absent, with a strong preference toward the formation of Ti-based phases. As such, a decrease in “A” element of MAX composition was observed. Previously, this occurred typically due to the evaporation of that element during manufacturing process and was a reason to use excess additions during syntheses^21^. The system with Ti_3_SiC_2_ precursor was the only one that created small amounts of carbides without boron due to the large amount of “A” element being missed. Only Cr_3_C_2_ phase was missing.

#### SEM/EDS analysis

**Fig. 2:**
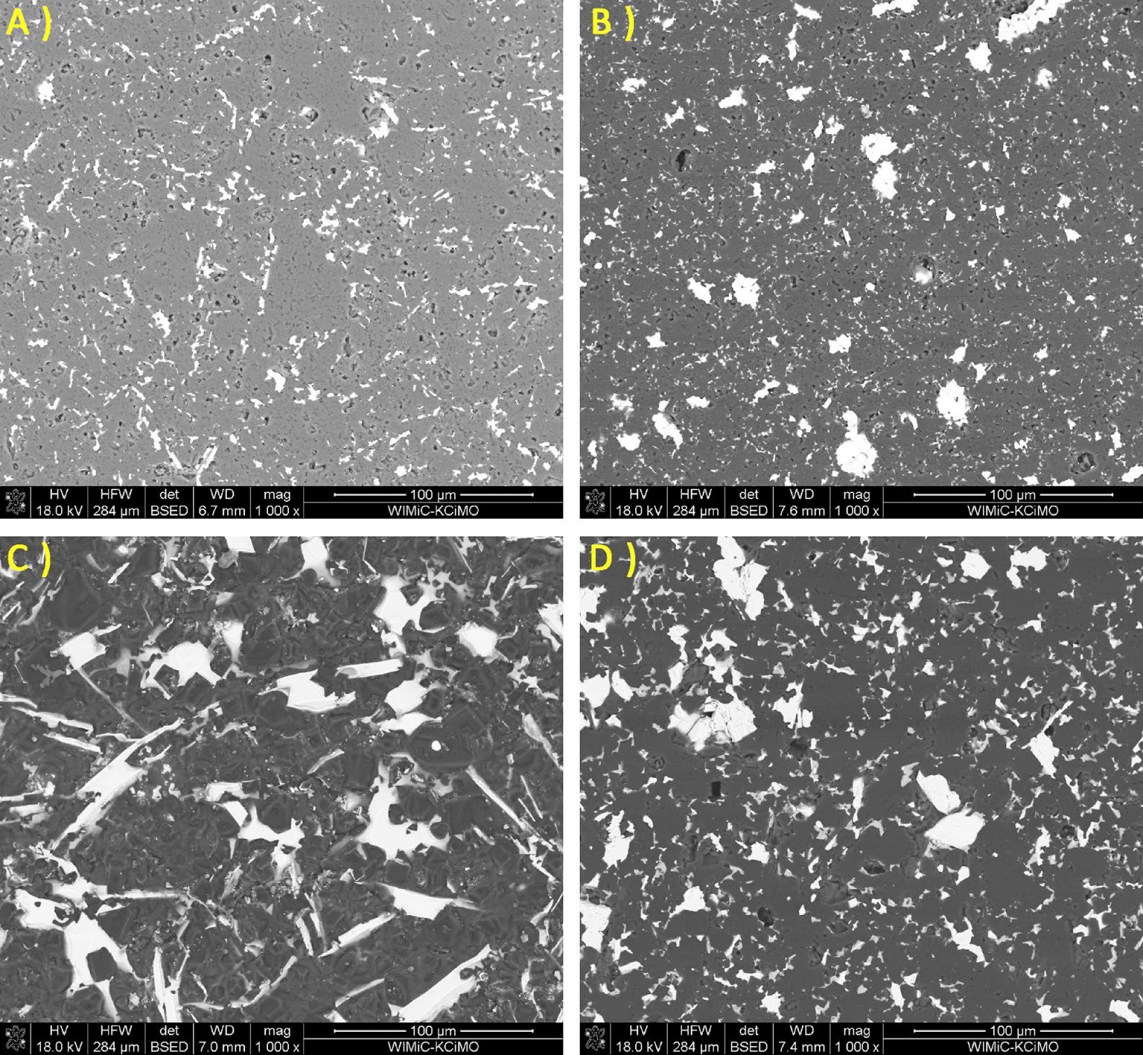
SEM images of the B_4_C_Ti_3_SiC_2_ sinters for the following Ti_3_SiC_2_ additions: (**A**) 5% (**B**) 10% (**C)** 15% (**D**) 20%.

**Fig. 3:**
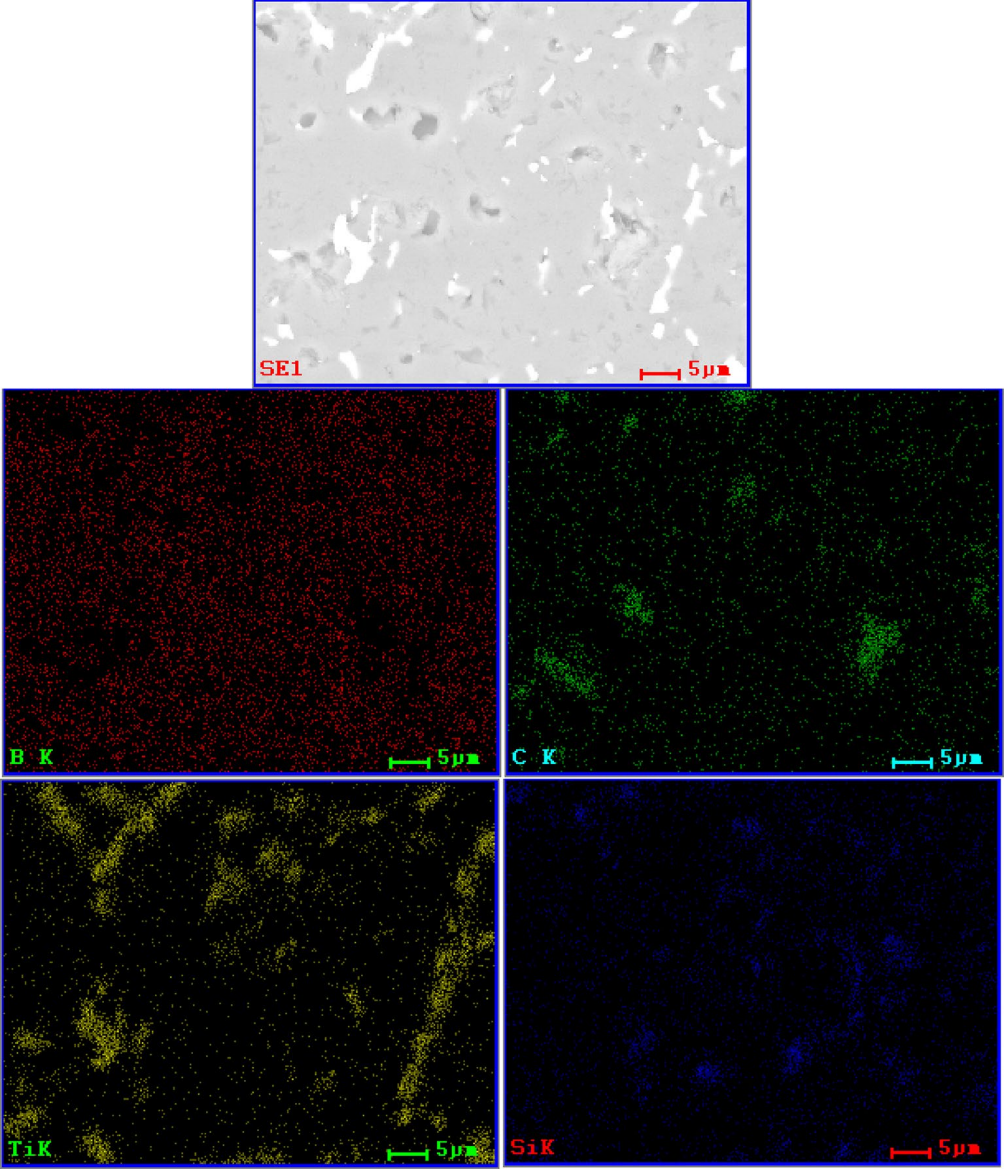
SEM/EDS elemental mapping of B_4_C_Ti_3_SiC_2__5 sample.

**Fig. 4:**
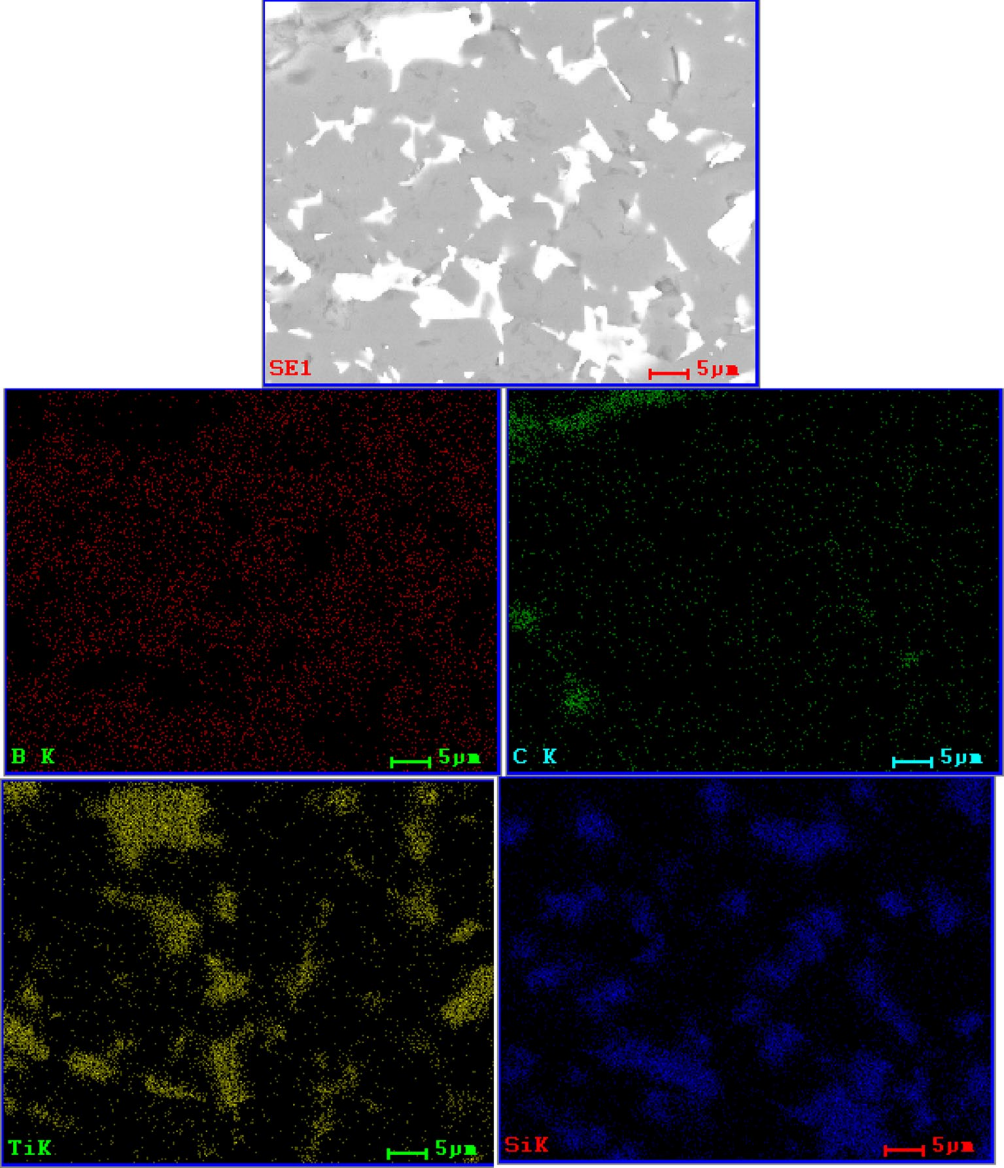
SEM/EDS elemental mapping of B_4_C_Ti_3_SiC_2__20 sample.

The observation of the material microstructure and further qualitative analysis were presented for each sample group in form of a comparison between each amount of added amount of MAX phase for a given group and followed by EDS spectra of the lowest and highest amount of given addition. In Fig. 2, the comparison for a B_4_C_Ti_3_SiC_2__X sample group was presented. It was observed that with an increase of Ti_3_SiC_2_ the size of some secondary phase, grains increased. While small defects were observed and presented mostly in the matrix and not at phase boundaries, those defects were largely considered to be coming from the polishing process of sample preparation. If the addition influenced the creation of such porosity, it should be present between bigger grains and the matrix. These would be observed in the sample group B_4_C_Ti_2_AlC_X and corresponded to the density observations. Meanwhile, the observed material of Ti_3_SiC_2_ additions was generally well sintered, which was especially visible for B_4_C_Ti_3_SiC_2__15. Sample of B_4_C_Ti_3_SiC_2__20 had notable defects appearing in the secondary phase. It again corresponded with the second described example of subchapter 4.2. It can also be noted that samples of B_4_C_Ti_3_SiC_2__5 and B_4_C_Ti_3_SiC_2__15 had similar sizes of secondary phase grains. Samples of B_4_C_Ti_3_SiC_2__10 and B_4_C_Ti_3_SiC_2__20 had a two-mode distribution of secondary phase grain size due to the potential partial agglomeration of the secondary TiC phase as shown later by the EDS. The EDS mappings for samples of 5 and 20 wt% addition of Ti_3_SiC_2_ are presented in Figs. 3 and 4 respectively. This analysis was limited to the presentation of dominating elements but with corresponding XRD analysis, phases could be named with a high degree of certainty. Such combined approach revealed that the secondary phase is composed mostly of phases containing Ti, which in this case would be TiB_2_ and TiC detected by the XRD as well as Si_5_C_3_ which was agglomerating with Ti-dominated secondary phases. Matrix was composed mostly of B-based, carbide phase, which was determined by the XRD analysis for each sample group to be B_4_C phase. Both B_4_C_Ti_3_SiC_2__5 B_4_C_Ti_3_SiC_2__20 sample possess good distribution of B in almost the whole microstructure, although for B_4_C_Ti_3_SiC_2__20 sample the amount of added Ti was sufficient to create small sections where B was not present.

**Fig. 5:**
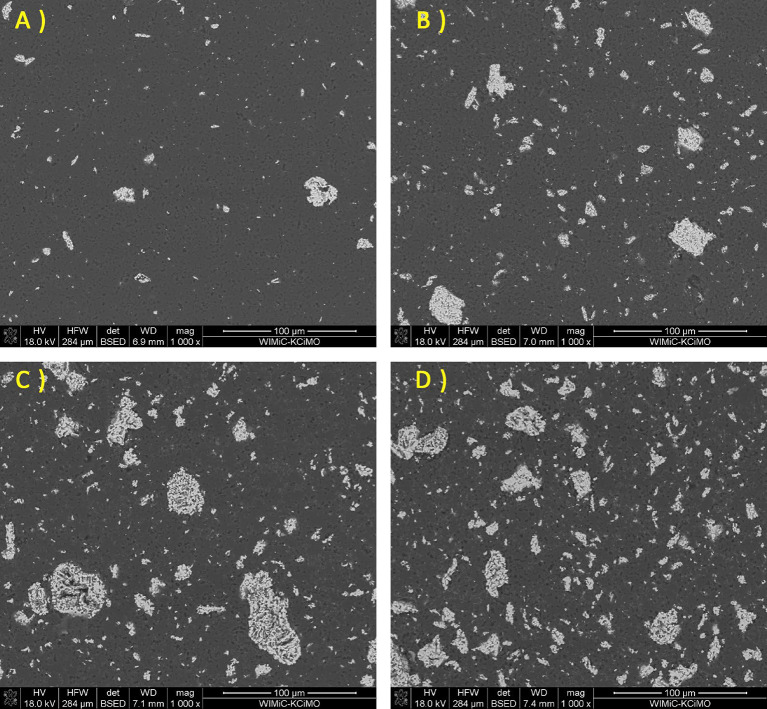
SEM images of B_4_C_Ti_2_AlC sinters for the following Ti_2_AlC additions: (**A**) 5% (**B**) 10% (**C**) 15% (**D**) 20%.

**Fig. 6:**
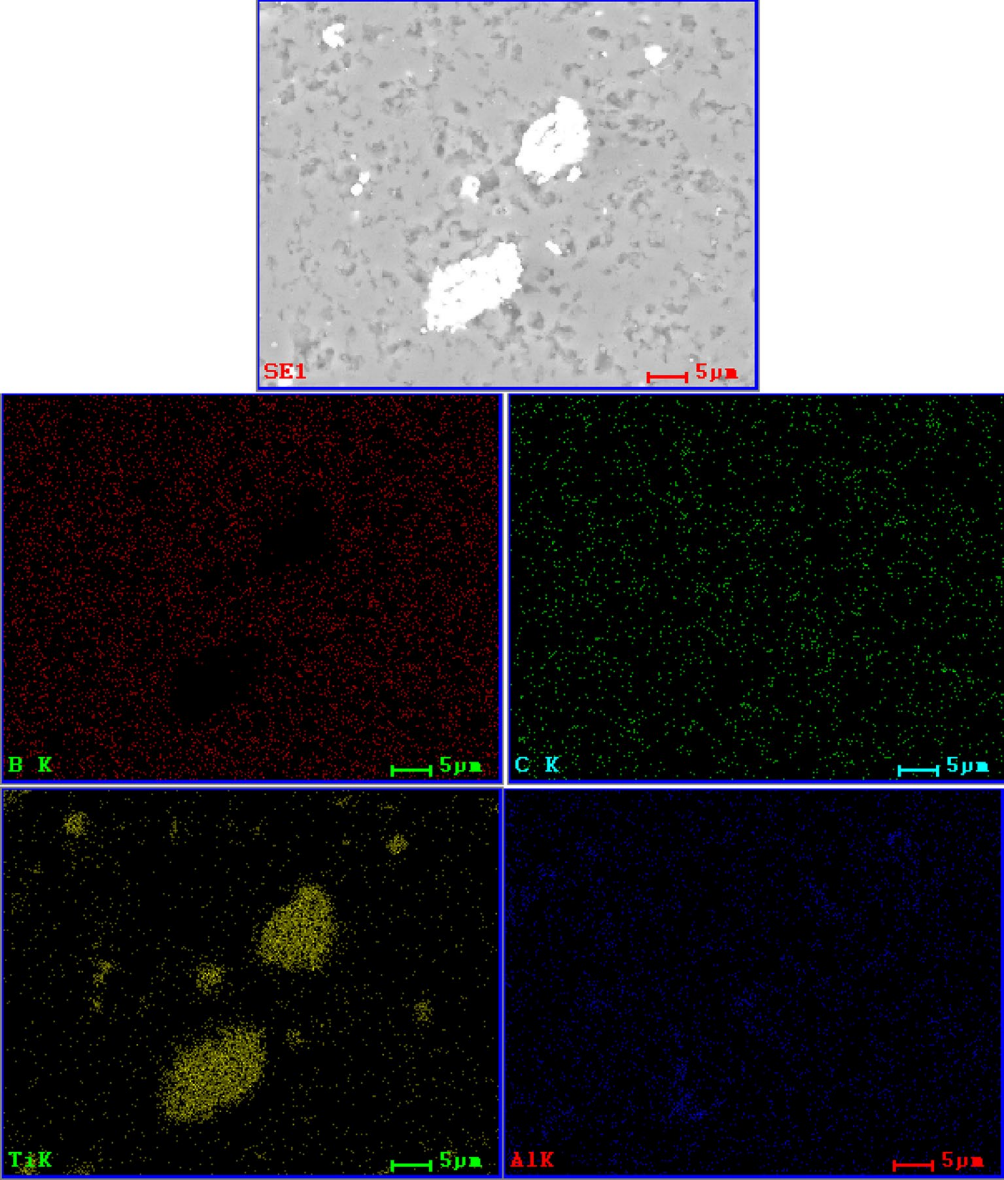
SEM/EDS elemental mapping of B_4_C_Ti_2_AlC_5 sample.

**Fig. 7:**
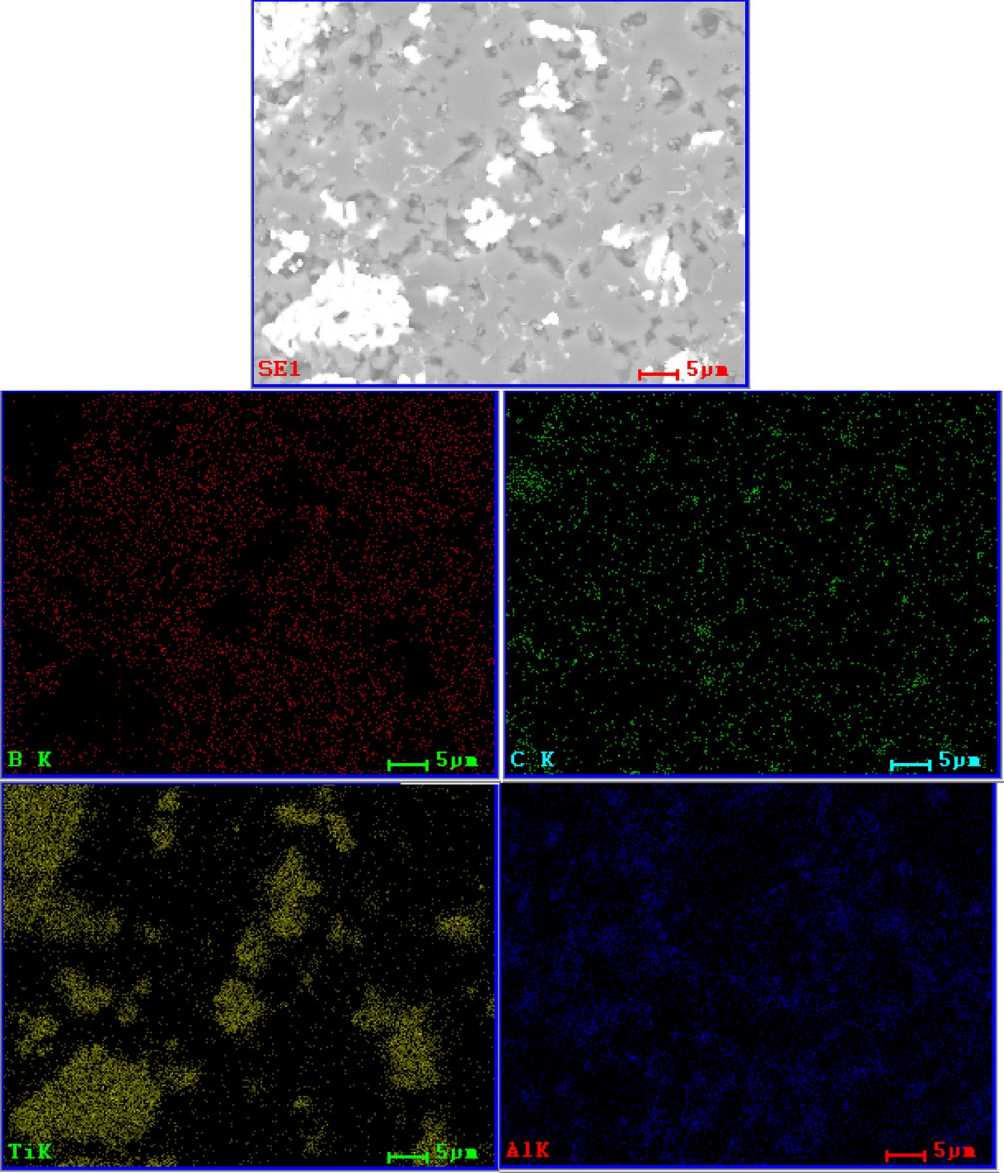
SEM/EDS elemental mapping of B_4_C_Ti_2_AlC_20 sample.

In Fig. 5 the comparison for a B_4_C_Ti_2_AlC_X sample group was presented. It was revealed that the distribution of secondary phase grains was very different than in samples with Ti_3_SiC_2_ additions. Their amount increases with increased addition, but the two-mode size distribution of those grains could be observed in a sample from each sample group, once again, due to the potential partial agglomeration of the secondary TiC phase. Moreover, grains were not elongated, but rather circular. The sintering process of samples with additions of Ti_2_AlC was imperfect, especially when compared to the previous sample group. Although matrix imperfections can be again noted as dominant, visible defects within the secondary phase were observed and more indicative of the sintering process. As indicated by EDS mapping (Figs. 6 and 7), the spaces between the grains were not composed of boron carbide for either the 5 or 20 wt% of added Ti_2_AlC. Although the method is limited to state that directly, it was declared based on the dominance of Ti or Al element and supporting analysis from subchapter 4.3.1. that does phases are most likely TiB_2_ and AlB_2_.

**Fig. 8:**
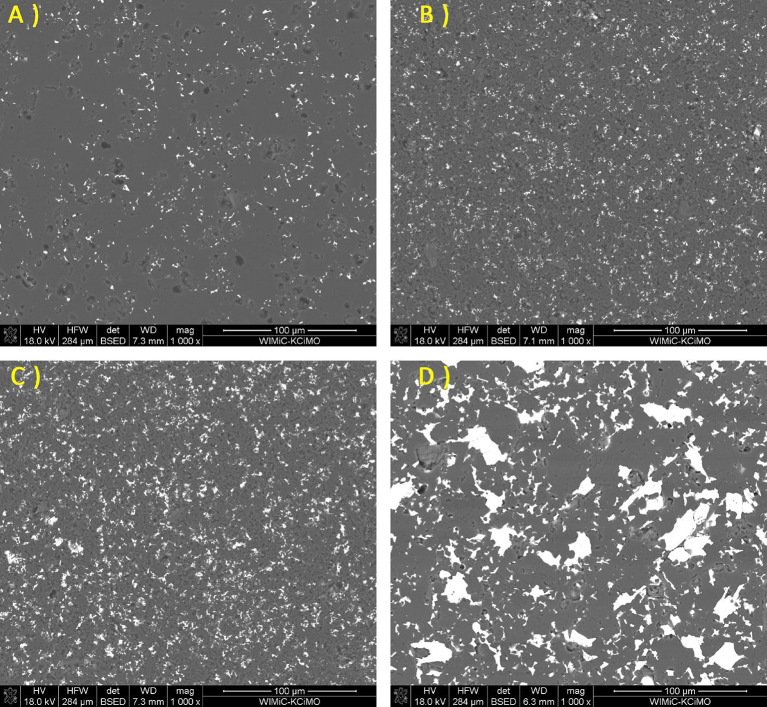
SEM images of B_4_C_Cr_2_AlC sinters for the following Cr_2_AlC additions: (**A**) 5% (**B**) 10% (**C**) 15% (**D**) 20%.

**Fig. 9:**
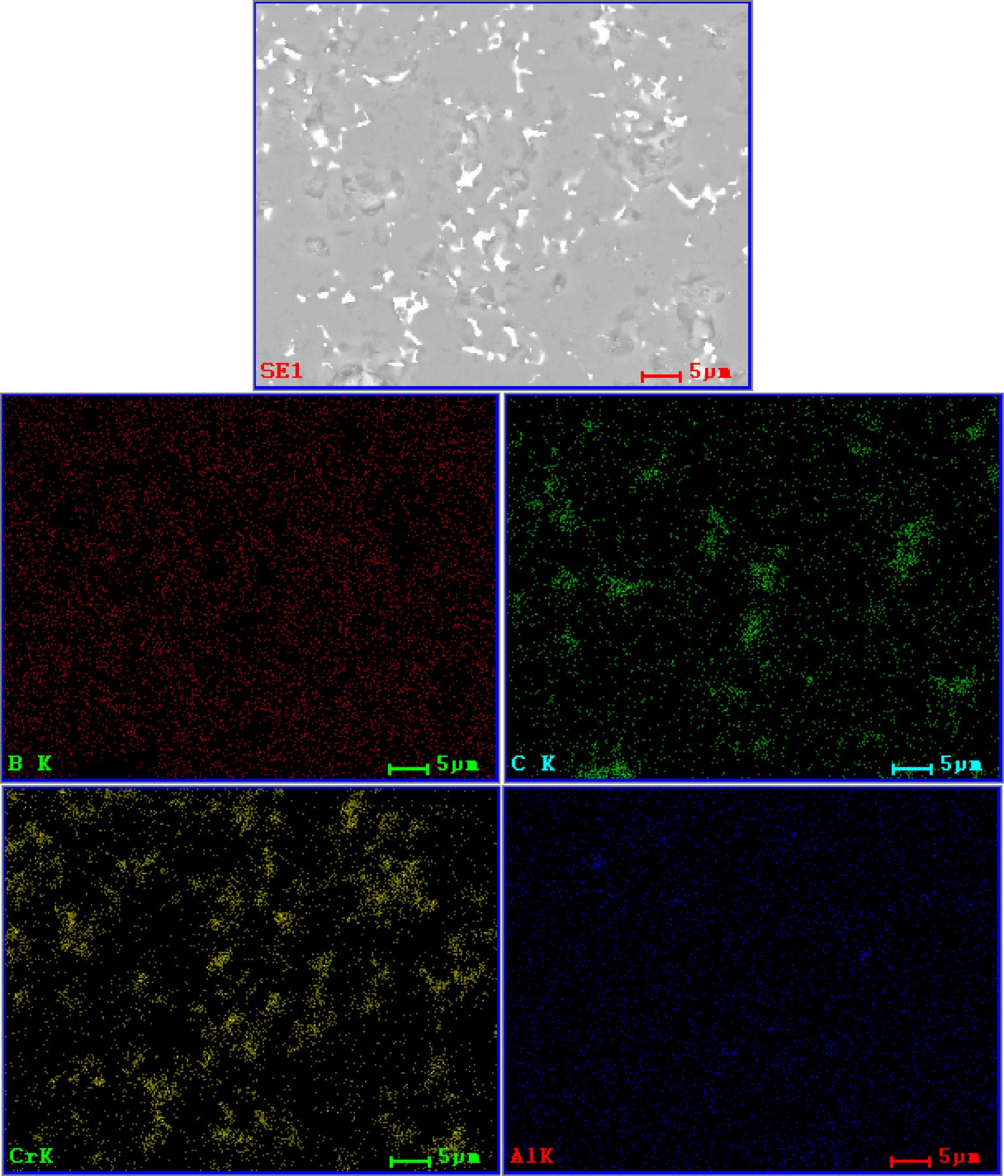
SEM/EDS elemental mapping of B_4_C_Cr_2_AlC_5 sample.

**Fig. 10:**
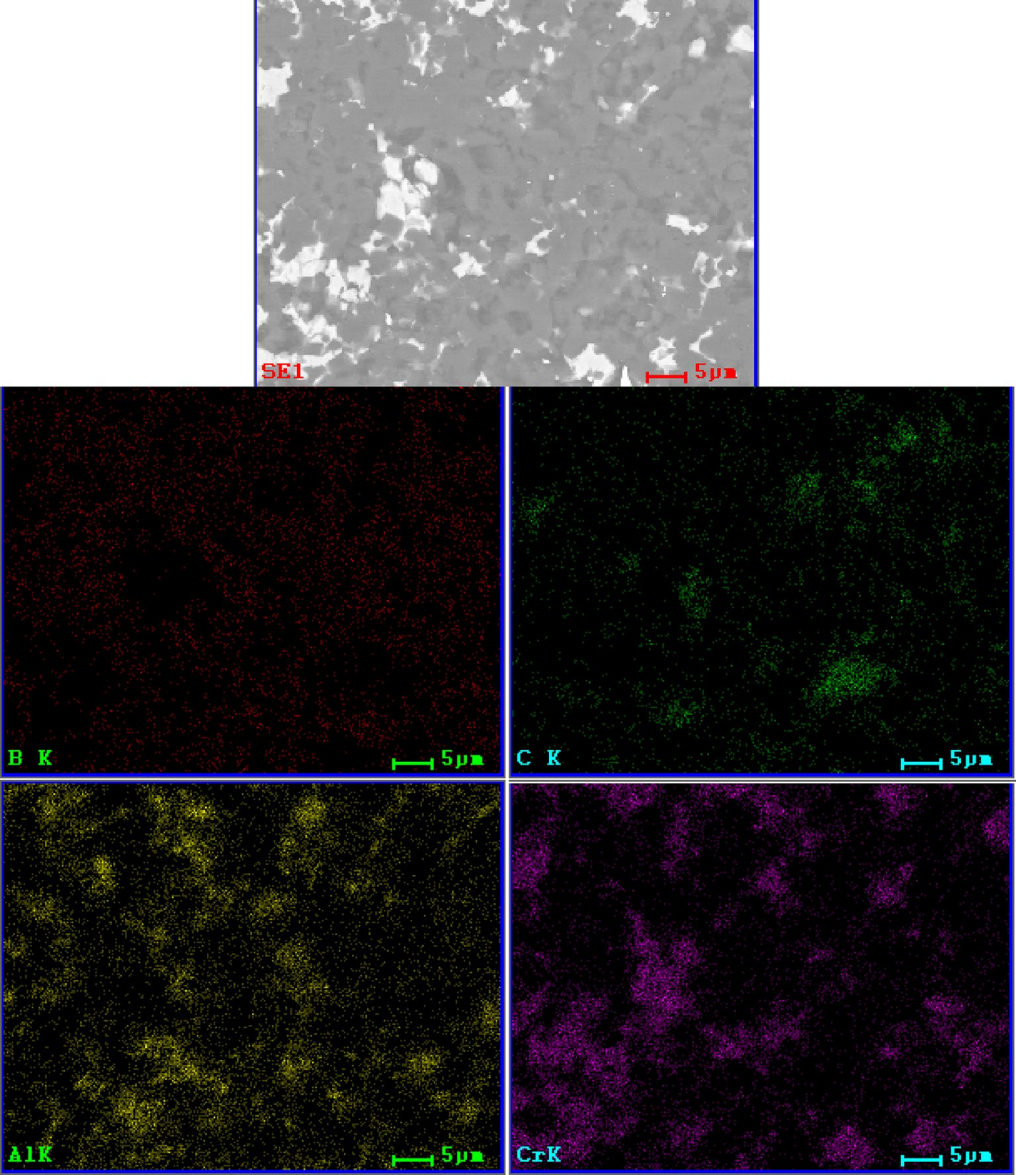
SEM/EDS elemental mapping of B_4_C_Cr_2_AlC_20 sample.

A microstructure comparison between samples of the B_4_C_Cr_2_AlC group was presented in Fig. 8. It could be noted, that up to 15 wt% of added Cr_2_AlC, secondary phase grains were not enlarging significantly. Only for samples containing 20 wt% of Cr_2_AlC addition, grains were of increased size. The B_4_C_Cr_2_AlC_20 sample was similar in its microstructure to the B_4_C_Ti_3_SiC_2__15 and B_4_C_Ti_3_SiC_2__20. Microstructures observations confirmed densification during sintering process with a very small amount of presented defects and clear phase boundaries (with the exception of additions reaching 20 wt%.). This corresponded to the analysis from subchapter 4.2, which indicated very small changes in the young modulus in-between samples of each group, while the measured density changed significantly for B_4_C_Cr_2_AlC_20 sample group, indicating observed change in the microstructure. Based on that, it can be discussed that the addition of Cr_2_AlC MAX phase had the least detrimental effect on the final sinter microstructure. In Figs. 9 and 10, the SEM/EDS analysis of those materials were presented.

As with the previous sample groups, the matrix and secondary phase can be easily distinguished for B_4_C_Cr_2_AlC_X samples. SEM images confirmed obtaining of the well-sintered composite materials with the low number of defects as the phases are intertwined with each other. However, their elemental distribution was not clearly related to the same matrix/secondary phase distribution in the microstructure (Figs. 9 and 10). Samples with additions of Cr_2_AlC possessed the highest amount of C clusters in their microstructures, but those clusters were not specifically related to any of the elements composing those materials. They were indicating that in those materials, the matrix possesses several stoichiometric variants of boron carbide as those clusters are often placed on the matrix. Although this cannot be stated by the usage of SEM/EDS mapping alone, it would be in pair with XRD analysis which had shown multiple peaks related to boron carbide phases.

### Vickers hardness and fracture toughness

**Fig. 11:**
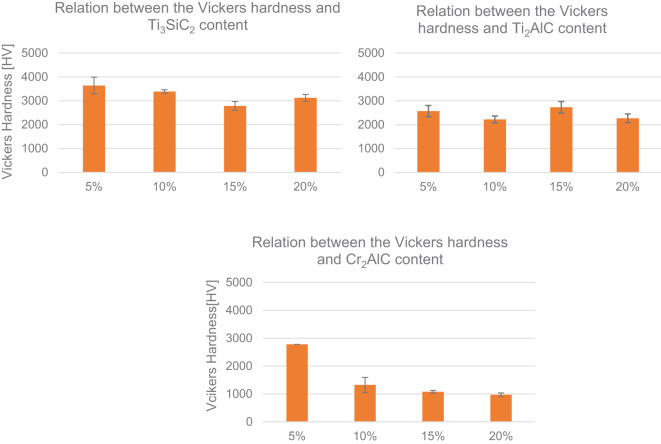
Comparison of Vickers hardness values measured for each sample group.

The evaluation of UHTCs mechanical properties was done based on the Vickers hardness (HV) and fracture toughness (K_IC_) parameters. The hardness measured for each sample group was presented in Fig. 11. In terms of that property, obtained multi-phase materials possessed very different values and relation to the amount of added MAX phase and its effect on the microstructure and overall composition of the obtained samples. When calculated to GPa, the lowest hardness was achieved by samples with Cr_2_AlC MAX phase precursor (9.5–27.3 GPa). In this group of obtained samples, hardness was largely decreased after the threshold of 5 wt% of addition was crossed which marks the amount of CrB_2_ secondary phase being of about 10–20% of the whole phase composition (Table 3). Its creation was perhaps not detrimental strictly by that presence alone as XRD analysis could indicate but also due to the several other stoichiometric variants of boron carbide that could crystalize as indicated by the SEM/EDS analysis. Importantly, the appearance of secondary phase agglomerates within the sample of 20% addition were not furthermore significantly decreasing or increasing the hardness, rather continuing the clear trend. Based on that, it can be argued that the lack of clear phase boundaries observed leads to the situation in which the propagation of microstresses is not blocked but rather carried over through the softer MAX phases, leading to the overall decrease, when the effect of addition can cross the threshold (here it was around 5% of the addition. That conclusion would however have to be confirmed by more thorough analysis, simultaneously narrowing down the threshold as the current step of 5% is too broad. Vickers hardness of samples with Ti_3_SiC_2_ and Ti_2_AlC had no clear relation to the amount of added MAX phase precursor but can be correlated to the porosity created within secondary phase grains as their size grows, based on the previous SEM/EDS analysis. For the B_4_C_Ti_3_SiC_2_ sample group, the decrease was noted up to 15 wt% of addition of secondary phase, while for the sample with higher content of Ti_3_SiC_2_ (20 wt%) hardness increase was observed. It can be related to the observed sudden larger agglomeration of the secondary phase with simultaneous obliteration of the phase boundaries in various places (Fig. 2). An effect which could be negated with further additions as the agglomeration could be large enough to clearly create the bi-modal distribution which was not that apparent within 5–10% samples and henceforth, increasing the overall hardness again. Similarly, the group with the addition of Ti_2_AlC had an observable variation based on the secondary phase behavior. Observed agglomeration of the secondary phase indicated strong presence of the TiC/TiB_2_ phases in the localized manner, which while tested could increase the hardness locally, leading to overall increase of mirohardness in the studies sample. However, it must be stated that for samples with Ti_2_AlC, the hardness change is simply variable and this method is not a reliable way of increasing this parameter of the original UHTC material. Moreover, those variable results could be affected by the differences of sintering temperatures, which varied due to the MAX phase additions and therefore greatly affected the phase boundaries of many different secondary phase iterations detected in phase anaslysis. The highest Vickers hardness was achieved for samples with Ti_3_SiC_2_ (27.3–35.7 GPa), which was the only batch comparable to the median values reported for commercially available, pure B_4_C ranging from 26 to 42 GPa. Calculated values for B_4_C_Ti_2_AlC were of the least difference in the sample group itself, ranging from 21.74 GPa for samples with the secondary phase addition of 10 wt% up to 26.75 GPa for samples with the secondary phase addition of 15 wt%.

**Fig. 12:**
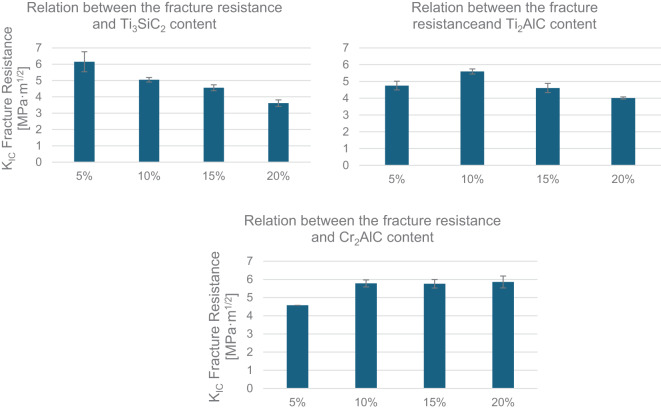
Compilation of fracture resistance measured for each sample group.

Figure 12 presents measurements of fracture resistance performed for each sample group. Samples created from B_4_C-Ti_3_SiC_2_ precursors had their fracture resistance decreased relatively to the increasing amount of secondary phase added. Samples of the B_4_C_Cr_2_AlC group showed a reversed trend. Finally, samples with the addition of Ti_2_AlC were improved by smaller additions of MAX phase precursors up to 10 wt% and further increase of that value was reducing their fracture resistance. The main explanation of the trends is the addition of the secondary phase initially blocks the crack propagations within the material. Composites created by additions of Ti_3_SiC_2_ and Ti_2_AlC MAX phases, initial increase of resistance is later decreased with growing wt% of both. This, as indicated by SEM/EDS analysis (subchapter 4.3.2.), is due to the agglomeration of the secondary phase by the creation of TiC/TiB_2_ which serves as the center of this process. While initially, this inclusion supports the main trend, by the growing addition of the MAX phases, the amount of noted defect was increasing, leading to the lower overall fracture resistance. However, as additions of Cr_2_AlC into the B_4_C was proven to be well integrated into the final microstructure of the material, the fracture resistance is not only initially improved but also gradually increases with further additions. This would be perhaps negated with further increasing the amount as the bi-modal distribution was eventually noted and the amount of defects could accumulate similarly to the samples with Ti_3_SiC_2_ and Ti_2_AlC MAX phases.

The highest K_IC_ factor was achieved for the B_4_C_Ti_3_SiC_2__5 sample (slightly above 6), while simultaneously the most brittle materials were also of the same sample group (B_4_C_Ti_3_SiC_2__20).

## Discussion

The mixture of MAX phases and B_4_C allows to create UHTCs composite-like structure applying significantly reduced sintering temperatures and preventing high final porosity in certain sample groups that can be chosen to be obtained by determining certain additions values. This reduction ranges up to 800 °C and therefore would be considered a low-temperature sintering^29^ of this material. Several obtained samples noted a simultaneous significant reduction of mechanical properties due to the secondary phase defects. Sample groups with Ti_2_AlC and Cr_2_AlC showed significantly reduced hardness and elasticity modulus. Fracture toughness (K_IC_) of obtained composite system was remarkably higher than that of a pure B_4_C. The improvement ranged from 33 to 100% when compared to both the literature^30^ and commercially available products^29^. This can be attributed to the tendency observed in XRD and SEM/EDS analyses. Boron carbide can crystalize very differently based on the conditions and as a matrix can adapt structurally to the secondary phases due to the room in its structure^6,33^. Applications of UHTCs in which smaller Young’s Modulus and hardness can be afforded, might greatly benefit from the fracture resistance of most of the obtained samples.

**Fig. 13:**
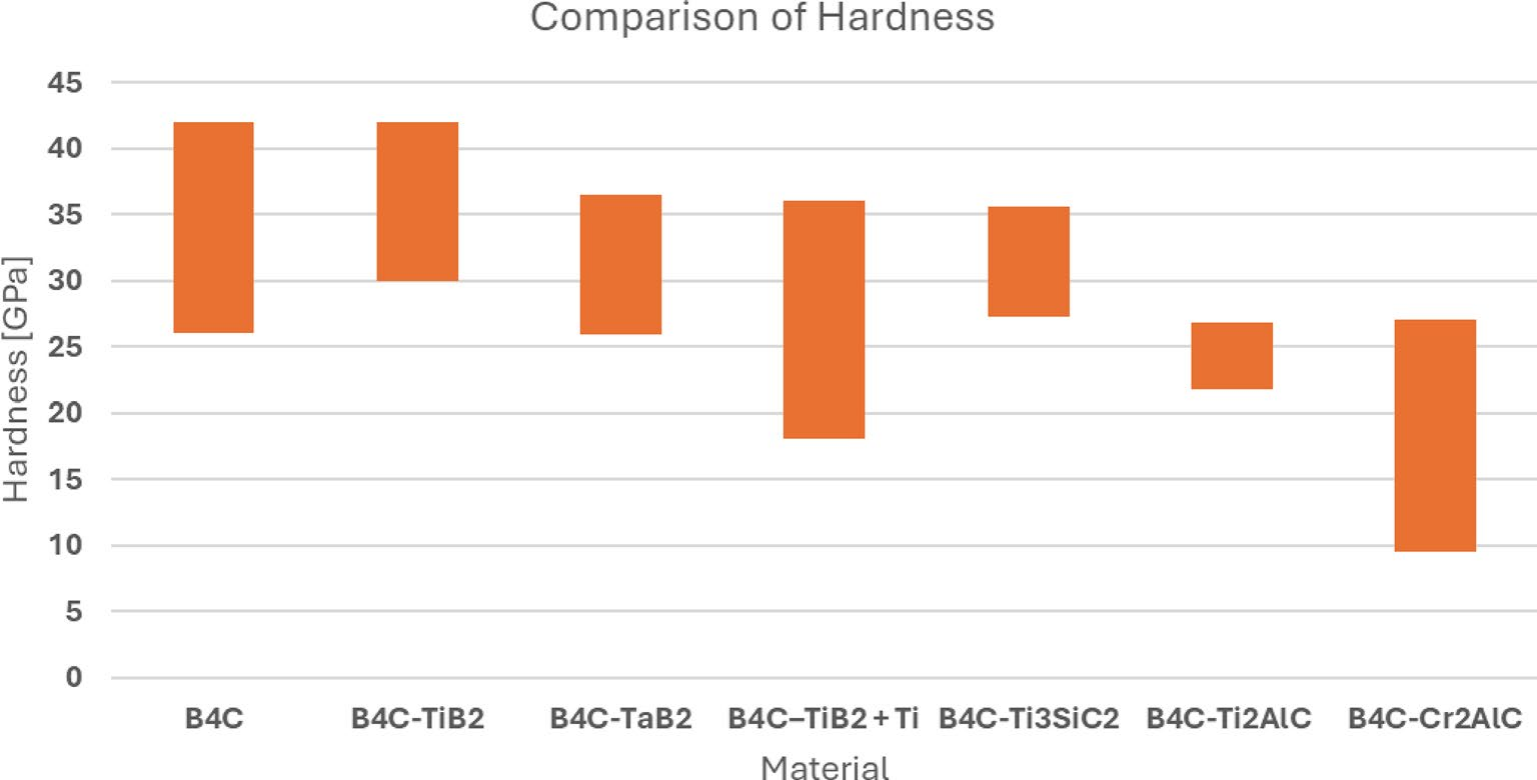
Compilation of hardness of obtained sample groups with UHTC materials serving similar purpose^35–38^.

**Fig. 14:**
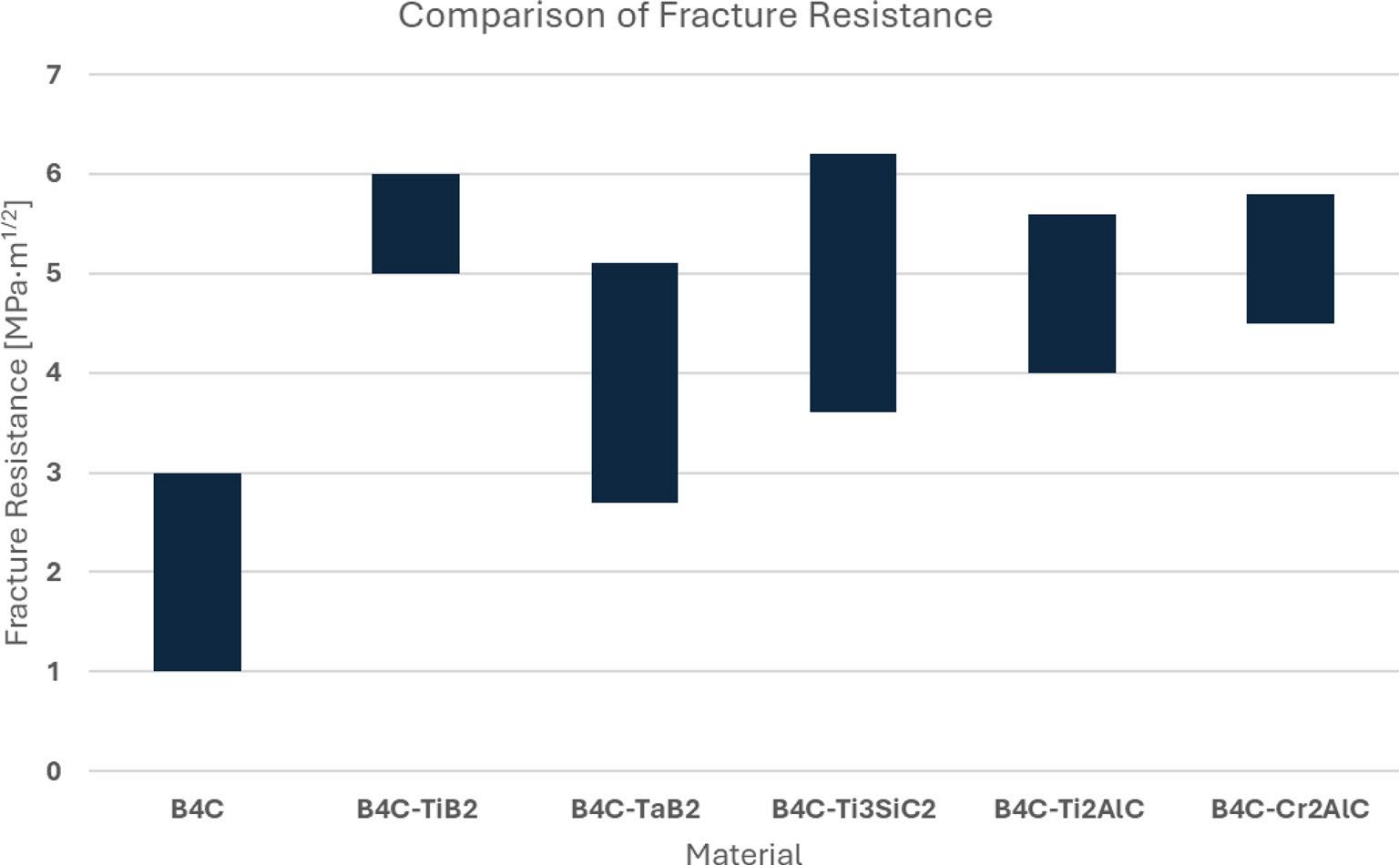
Compilation of fracture resistance of obtained sample groups with UHTC materials serving similar purpose^35–38^.

While comparison to pure B_4_C might yield unfavorable conclusion, it is also important to set together obtained materials with similar UHTC systems that use Ti-based compounds to improve industrial potential by reducing sintering temperatures and improving fracture resistance. To evaluate how the obtained materials stand up within such group, two comparisons: of hardness and fracture resistance were prepared (Figs. 13 and 14).

The comparison reveals that hardness decrease among similar materials is often observable. Solodkyi et al. indicates that this decrease often occurs due to the Ti-rich regions having significantly less punctual hardness than the boron carbide eutectic particles^37^. When averaged, obtained materials reinforced by introduction of MAX phases, due to the shown distribution of many secondary phases, demonstrate significant decrease, but the B_4_C_Ti_3_SiC_2_ sample group is in pair to the other solution. Where significant improvement, especially over UHTC with TaB_2_ can be observed is fracture toughness. Every studies sample group of this research shown not only significant improvement over reference material but also in comparison to similar UHTC materials.

The uncontrolled evaporation of Si during the Ti_3_SiC_2_ obtaining processes is typical technological problem^21^. In obtained samples, this presented itself in relatively small amount of Si-based secondary phase, especially when compared to the Al-based and Cr-based secondary phases of other sample groups. At the same time, the B_4_C_Ti_3_SiC_2_ sample group exhibited the best properties of all described systems and can be studied further in terms of their properties, application potential, and advanced obtaining technologies. They maintained high elasticity modulus and high hardness, comparable to the pure B_4_C with the simultaneous decrease in sintering temperatures. Although this reduction was lower than in the case of other systems, it is still significant to potential technologies. Moreover, the resulting improvement of K_IC_, which was the highest of the three proposed systems cannot be neglected. As such, those materials stand as potentially unique UHTCs of highly improved fracture resistance. This could find several applications in modern industries, provided that in the future their elevated temperature performance would be evaluated in an extensive study. It would also be important to extensively study their thermal properties and shear resistivity.

## Conclusions

- Results of performed experiments showed that among MAX phases used in presented work as addition to boron carbide only the Ti_3_SiC_2_ phase gave finally composite material showing prospective properties.
- The conducted study can be concluded with the following points:
- Addition of MAX phase precursors allowed to manufacture the multi-phase well-sintered UHTCs in significantly reduced sintering temperatures and with lower density than pure B_4_C, and therefore lower weight of potential products containing high amount of boron carbide phase.
- Combined SEM/EDS analysis revealed that samples with Cr_2_AlC precursors were well sintered, while samples with Ti_3_SiC_2_ and Ti_2_AlC had added defects observed within secondary phase.
- The B_4_C_Ti_3_SiC_2_ sample group exhibited a strength comparable to the median of the results reported for pure B_4_C, material which requires sinter additives and therefore making Ti_3_SiC_2_ additive potentially useful.
- Addition of all used MAX phase precursors led to the remarkable improvement of fracture toughness in comparison to the pure B_4_C. In principle, secondary phases blocked the crack propagation. However, with their growing additions, defects could emerge. This tendency was especially noticeable by comparing samples with Cr_2_AlC precursors to the Ti_3_SiC_2_ and Ti_2_AlC precursors.
- Samples of B_4_C_Ti_3_SiC_2_ combined good mechanical properties of B_4_C, with lower sintering temperatures and highly improved K_IC_. Provided that extensive studies will be done, this system is potentially applicable in the industry.

## Data Availability

The datasets used and/or analysed during the current study available from the corresponding author on reasonable request.
